# Tanshinone IIA Increases the Bystander Effect of Herpes Simplex Virus Thymidine Kinase/Ganciclovir Gene Therapy via Enhanced Gap Junctional Intercellular Communication

**DOI:** 10.1371/journal.pone.0067662

**Published:** 2013-07-04

**Authors:** Jianyong Xiao, Guangxian Zhang, Pengxiang Qiu, Xijuan Liu, Yingya Wu, Biaoyan Du, Jiefen Li, Jing Zhou, Jingjing Li, Yuhui Tan

**Affiliations:** 1 Department of Biochemistry, Guangzhou University of Chinese Medicine, Guangzhou, China; 2 Department of Pathology, Guangzhou University of Chinese Medicine, Guangzhou, China; Albert Einstein College of Medicine, United States of America

## Abstract

The bystander effect is an intriguing phenomenon by which adjacent cells become sensitized to drug treatment during gene therapy with herpes simplex virus thymidine kinase/ganciclovir (HSV-tk/GCV). This effect is reported to be mediated by gap junctional intercellular communication (GJIC), and therefore, we postulated that upregulation of genes that facilitate GJIC may enhance the HSV-tk/GCV bystander effect. Previous findings have shown Tanshinone IIA (Tan IIA), a chemical substance derived from a Chinese medicine herb, promotes the upregulation of the connexins Cx26 and Cx43 in B16 cells. Because gap junctions are formed by connexins, we hypothesized that Tan IIA might increase GJIC. Our results show that Tan IIA increased GJIC in B16 melanoma cells, leading to more efficient GCV-induced bystander killing in cells stably expressing HSV-tk. Additionally, in vivo experiments demonstrated that tumors in mice with 10% HSV-tk positive B16 cells and 90% wild-type B16 cells became smaller following treatment with the combination of GCV and Tan IIA as compared to GCV or Tan IIA alone. These data demonstrate that Tan IIA can augment the bystander effect of HSV-tk/GCV system through increased gap junction coupling, which adds strength to the promising strategy that develops connexins inducer to potentiate the effects of suicide gene therapy.

## Introduction

Gene therapy using the herpes simplex virus thymidine kinase (HSV-tk) gene has gained prominence as a potential therapeutic modality for inhibiting cell proliferation in the context of tumors [1], [2]. This strategy uses the ability of the HSV-tk gene to sensitize tumor cells to the antiviral drug ganciclovir (GCV). The encoded HSV-tk gene activates the GCV into a cytotoxic metabolite to inhibit tumor cell survival [3]. Present delivery systems, such as adenoviruses or retroviruses, have proven unable to reach the total cancer population [4], [5]; consequently, gene therapy trials have often been considered inefficient in cell targeting and have demonstrated a low overall success rate [4]. However, the addition of GCV kills both cells expressing HSV-tk and nearby tumor cells that do not express it. This phenomenon of increasing killing of adjacent tumor cells is termed the “bystander effect” [6]. Because not all of the tumor cells need to be directly targeted, the occurrence of the bystander effect in HSV-tk/GCV therapy may represent an important therapeutic opportunity. The mechanism of the bystander effect in the HSV-tk/GCV system may include a number of factors; however, compelling evidence demonstrates that gap junctional intercellular communication (GJIC) is directly involved [7], [8], [9], [10]. GJIC is thought to function by allowing the passive diffusion of an activated metabolite to neighboring cells, thereby enabling the drug to target a greater proportion of the cell population.

Gap junctions are formed by connexins [11], a family of homologous proteins that directly link the cytoplasms of adjacent cells to allow the passage of ions and small molecules of up to 1 kDa. Connexins can act as tumour suppressor genes [12]. Changes in the connexin expression, in particular the loss of connexin 43, may result in a reduction or a loss of gap junctional activity, which contributes towards melanoma progression [13]. GJIC induction resulting from transfection of connexins leads to a decreased rate of proliferation, increased differentiation, and reversal of the cell-transformation phenotype [14], [15]. Cx26, Cx30 and Cx43 are prevailing connexins in skin tissue that are often differentially expressed in skin tumors [16], [17], [18]. Transfection or transduction of Cx26 and Cx43 results in increased GJIC, and consequently leads to an increased bystander effect seen in suicide gene therapy [7], [19]. Alternatively, a number of classes of chemicals, including gemcitabine [20], cAMP [21], and retinoids [22], have been reported to increase Cx26 and Cx43 and, subsequently, GJIC.

Tanshinone IIA (Tan IIA) is a chemical substance extracted from the roots of dashen, a highly valued herb used in traditional Chinese medicine to treat cardiovascular diseases [23]. It is reported to possesses anti-inflammatory activities [24] and anti-oxidant properties [25] and most recently has been shown to possess anticancer activities both in cell culture and animal carcinogenesis models [26], [27]. Previously, Tan IIA was reported to restore Cx43 protein by depressing the elevated miR-1 level in ischemic and hypoxic cardiomyocytes [28]. Our current study confirmed that Tan IIA can upregulate Cx26 and Cx43 expression and increase GJIC in B16 melanoma cells. Therefore, we hypothesized that treatment of tumor cells with Tan IIA could augment the bystander effect of the HSV-tk/GCV system and result in improved tumor cell killing by enhancing GJIC. To test this hypothesis, we examined the effect of Tan IIA on bystander-mediated cell killing of B16 cells in vitro and in vivo. The results demonstrate Tan IIA may be a promising new approach to improve responses in gene therapy utilizing the HSV-tk/GCV system.

## Materials and Methods

### Chemicals and Reagents

Tan IIA (>98% pure) was purchased from Sigma Chemical Co. (St. Louis, MO, USA). GCV was obtained from InvivoGen Co. (San Diego, CA, USA) and dissolved in PBS before use. Calcein AM and DiI were obtained from Molecular Probes, Inc. (Eugene, OR, USA).

### Cell Lines and Cell Culture

The murine malignant melanoma cell line B16 (Wild-Type, WT) was obtained from American Type Culture Collection. The stable HSV-tk-expressing B16 cell line, B16 cells stably expressing HSV-tk and enhanced green fluorescent protein (EGFP), and B16 cells stably expressing red fluorescent protein (RFP) were established in our laboratory. Cells were cultured in RPMI 1640 supplemented with 10% Fetal Bovine Serum (FBS) and 100 U/mL penicillin and streptomycin.

### Animals

SPF-class C57BL/6J mice (equivalent numbers of males and females, weighing 18–22 g) were purchased from the laboratory animal center at Sun Yat-Sen University and maintained in the animal facility at Guangzhou University of Chinese Medicine. All protocols were approved by the Animal Experimentation Ethics Committee of Guangzhou University of Chinese Medicine in compliance with the recommended NIH guidelines for care and use of animals for scientific purposes.

### Immunoblot Analysis of Connexin Expression

B16 cells were seeded (1×10^4^ cells/well) in quadruplicate into 6-well plates. After 24 h, the medium was removed and replaced with complete medium containing Tan IIA (0, 5, 10 and 20 µM, respectively). Following three days of consecutive administration of Tan IIA, cells were washed twice with PBS and lysed in RIPA buffer (0.25 m Tris-HCl pH 6.8, 8% SDS, 1 mM phenylmethylsulfonyl fluoride, 10 mg/mL aprotinin, 1.0 mg/mL leupeptin). Total cell extracts were subjected to 10% SDS-PAGE, and immunoblotted using anti-Cx26 (CX-12H10; Zymed, San Francisco, CA, USA ), anti-Cx30 (71-2200; Zymed, San Francisco, CA, USA) anti-Cx43 (71-0700; Zymed, San Francisco, CA, USA) or anti-actin (ms-1295-po, NeoMarkers, CA, USA).

### Functional Assay of GJIC

GJIC function of was examined by the fluorescent dye transfer method as described by Goldberg with minor modification [29]. Briefly, 1×10^4^ B16 cells per well were seeded into 6-well plates. When the cells adhered, medium was supplemented with Tan IIA. After Tan IIA treatment for 18 h, the donor cells were digested in a single cell suspension with trypsin and then pre-loaded with two fluorescent dyes in serum-free medium: DiI and Calcein AM. DiI (a lipophilic red fluorescent dye) does not pass through gap junctions, while Calcein (a transferable green dye) passes readily between functionally coupled cells in a gap junction-dependent manner. Pre-loaded donor cells were mixed with unlabeled cells (recipient cells) at a 1∶100 ratio and at high density to maximize cell-cell contact. Flow cytometry analysis was performed after co-culturing for 6 h in the presence of Tan IIA to determine the level of GJIC. The excitation wavelength used was 488 nm, and the emission wavelengths for Calcein and DiI were 525 nm and 575 nm, respectively. Extent of dye coupling is defined by the percentage of recipient cells that received Calcein from donor cells (which is only possible through gap junctions). Four doses of Tan IIA treatment (0, 5, 10 and 20 µM) were used. The entire experiment was repeated three times.

### Analysis of Bystander Effect by Mixing of Stable HSV-tk-EGFP and RFP Cell Lines

Stable HSV-tk-EGFP-expressing B16 cells were mixed with stable RFP-expressing B16 cells at a ratio of 1∶1. The mixtures were then seeded at 3×10^3^ cells per well in 96-well culture plates. After the cells adhered, the medium was removed and replaced with complete medium or complete media containing Tan IIA (10 or 20 µM). Following 24 h, the cells were approximately 20%–30% confluent with most cells having visible contact with adjacent cells. Cells were treated with or without GCV at a concentration of 15.7 µM for 48 h in the presence of Tan IIA. The treated cells were then observed by fluorescence microscopy. The aggregation of red fluorescence indicated the apoptosis of stable RFP-expressing cells, mainly caused by the bystander effect of the HSV-tk suicide gene. The apoptosis of RFP cells was also analyzed by flow cytometry with annexin V stain. The apoptotic cells were the RFP and annexin V double-positive. The experiment was repeated three times.

### Assessment of the Effects of Tan IIA and GCV on the Growth of Mixed Cells

The stable HSV-tk-expressing B16 cell line was mixed with WT B16 cells at a ratio of 1∶9. As a measure of cell viability, the mixtures were then seeded at 3×10^3^ cells per well in 96-well tissue culture plates. After the cells adhered, the medium was removed and replaced with complete medium without or with Tan IIA (5, 10, 20 and 40 µM). Following 24 h, the cells were treated without or with GCV at concentration of 15.7 µM for 48 h in the presence of Tan IIA and then the medium was removed and MTT [100 µL (5 g MTT per 1 L serum-free medium)] was added to the cells, which were then cultivated for a further 4 h. After removing the supernatant, 100 µL/well of DMSO was added to the cells, which were then agitated for 15 min. The absorbance was measured at 570 nm by an ELISA plate reader. The inhibition rate = 1 − survival rate = (1 − OD_treatment_/OD_control_) ×100%. Each assay was repeated three times. For cell cycle analysis, 1×10^4^ mixed cells per well were seeded into 6-well plates. After the cells adhered, the cells were subjected to the drug treatment as above. Then, the cells were washed twice with PBS and then centrifuged. The pellet was fixed with 70% ethanol at 4°C, and then ethanol was washed away and the cells were treated with 40 mg/mL propidium iodide and 0.1 mg/ml RNase (Boehringer, Germany) for 30 min at 37°C. The cells (1×10^4 ^each group) were analyzed and the DNA content was measured using a FACStar cytofluorometer (BD Biosciences) equipped with an argon-ion laser at 488 nm.

### In vivo Experiments

A 1∶9 mixture of HSV-tk B16 cells to WT B16 cells was selected to test the impact of Tan IIA on the bystander effect in vivo. Mixed cells (5×10^6^) were injected i.p. into the right flanks of C57BL/6J mice. The mice were randomized to four groups (n = 20 mice per group): a saline control group; a group treated with GCV (100 mg/kg/day); a group treated with Tan IIA (100 mg/kg/day); and a group treated with GCV and Tan IIA. Treatment with Tan IIA was initiated on the next day of tumor injection and maintained for 14 days. Eight days after tumor injection, GCV (100 mg/kg/day) treatment was began and was continued over a seven day course.

### Statistics

For in vitro mixing studies, the percentage of cell survival was expressed as the mean ± standard deviation of the mean of 4 replicate wells for each mixture of cells. The means for each mixture of cells were compared utilizing analysis of variance (ANOVA). When significant differences were found, Fisher’s test was used to make comparisons among groups. *P*<0.05 was considered statistically significant. *significance compared with control; ^#^significance compared with Tan IIA or GCV treatment alone. Statistical significance is indicated in the graphs by one symbol for p<0.05, two symbols for p<0.01. Tumor weights were also expressed as the mean ± standard deviation of the mean and compared utilizing ANOVA.

The combination effect of drugs was assessed with Q value using Zheng-Jun Jin’s method of [30], Q = E_AB_/[E_A_+E_B_ (1 −E_A_)] (E_A_, E_B_ and E_AB_ indicated the effects of drug A, drug B and the combination of two drugs). According to "Q" value, the combination effect between two drugs can be classified as an antagonistic effect (Q <0.85), an additive effect (0.85< Q <1.15), or a synergistic effect (Q >1.15).

## Results

### Tan IIA Increases the Expression of Cx26 and Cx43 in B16 Melanoma Cells

**Figure 1:**
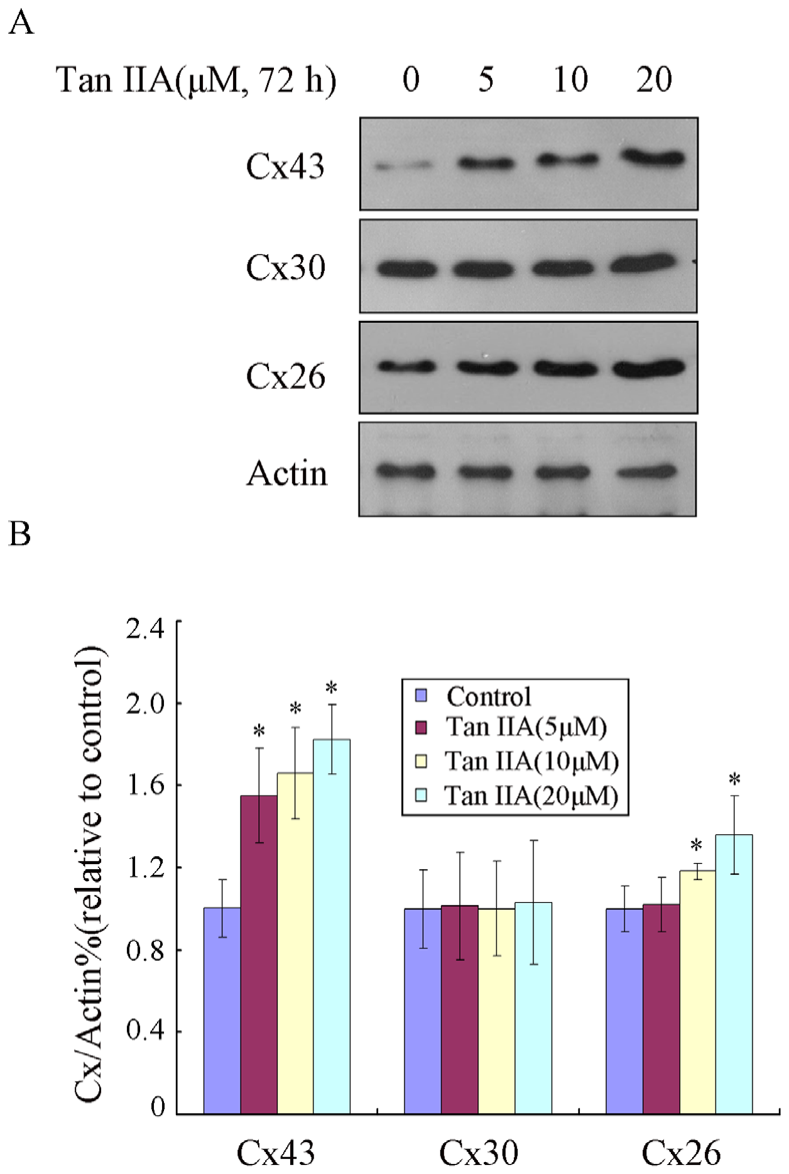
Tan IIA treatment of B16 cells results in the upregulation of Cx26 and Cx43 proteins. B16 cells were treated with Tan IIA (0, 5, 10 or 20 µM) for 72 h. (A) Immunoblotting was performed using antibody against Cx26, Cx30 and Cx43. Actin was also tested as a loading control. (B) Relative quantification of the immunoblotting results as calculated by gray scanning (*p<0.05). The results shown are representative of three independent experiments.

Tan IIA was reported to restore Cx43 protein by depressing the elevated miR-1 level in ischemic and hypoxic cardiomyocytes [28]. To further ascertain whether Tan IIA affects the expression of connexins in B16 cells, we examined its effect on the expression of Cx26, Cx30 and Cx43, which are the most predominant gap junction proteins in melanoma cell lines. B16 cells were cultured and treated with Tan IIA (0, 5, 10 and 20 µM) for 72 h. Immunoblot results indicated that the expression of Cx26 and Cx43 was markedly upregulated (*p<0.05), while the expression of Cx30 was unchanged after Tan IIA treatment (Figure 1). The same results were also observed after Tan IIA treatment for 24 h (Figure S1). Therefore, exposure of B16 cells to Tan IIA selectively upregulated the expression of the Cx26 and Cx43 connexins.

### Tan IIA Enhances Intracellular Communication as Assessed by Fluorescent Dye Transfer Experiments

**Figure 2:**
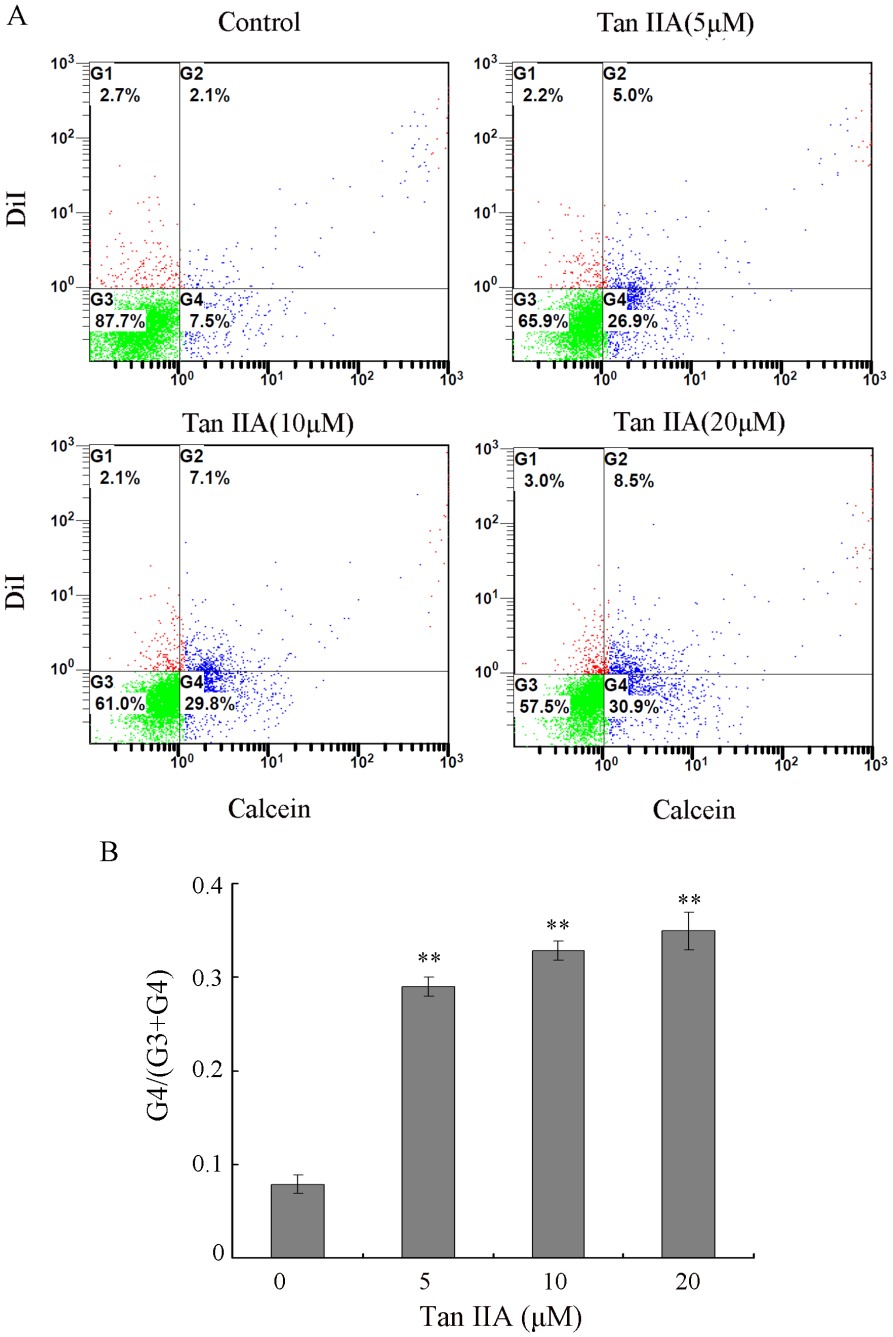
Tan IIA promotes GJIC of B16 cells, as measured by fluorescent dye transfer. B16 cells were left untreated (control) or treated with Tan IIA (5, 10 or 20 µM) for 18 h. Donor cells were pre-loaded with two fluorescent dyes: DiI and Calcein AM. A 1∶100 mixture of labeled donor cells and unlabeled recipient cells was co-cultured for 6 h in the presence of Tan IIA followed by flow cytometry analysis. (A) Flow cytometric analysis for fluorescent dye transfer.G1: Cell populations positive for DiI; G2: Cell populations positive for both DiI and Calcein; G3: Double negative cells; G4: Cell populations positive for Calcein only. (B) Quantification of three independent experiments. The G4/(G3+G4) of experimental groups was higher than that of the control, indicating more efficient cell-to-cell spread of Calcein after Tan IIA treatment. **p<0.01 compared with control.

Connexins are known to have important roles in the formation of gap junctions. Given that Tan IIA treatment resulted in the upregulation of Cx26 and Cx43, we sought to determine whether Tan IIA may also affect the formation of gap junctions in B16 cells. We used a fluorescent dye transfer experiment to assess GJIC following Tan IIA treatment. Figure 2 shows the results of this assay for B16 cells treated with Tan IIA (0, 5, 10 and 20 µM). G4 indicates the recipient cells that received Calcein from donor cells through gap junctions; G3 indicates the Calcein-negative recipient cells. The ratio of B16 cell numbers in quadrants G4 (Calcein-positive) and [G3 (Calcein-negative cells) plus G4] was used to evaluate the transfer of Calcein as an indication of GJIC function. As seen in Figure 2B, The G4/(G3+G4) of control was 0.08. In comparison, after various concentration of Tan IIA treatment (0, 5, 10 and 20 µM), the ratio of G4 to (G3+G4) was 0.29, 0.33 and 0.35, respectively (p<0.01). The results demonstrate that treatment with Tan IIA significantly enhances Calcein transmission in a dose-dependent manner, which suggests that GJIC in the B16 cell line is enhanced by Tan IIA treatment.

### In vitro Analysis of the Bystander Effect

**Figure 3:**
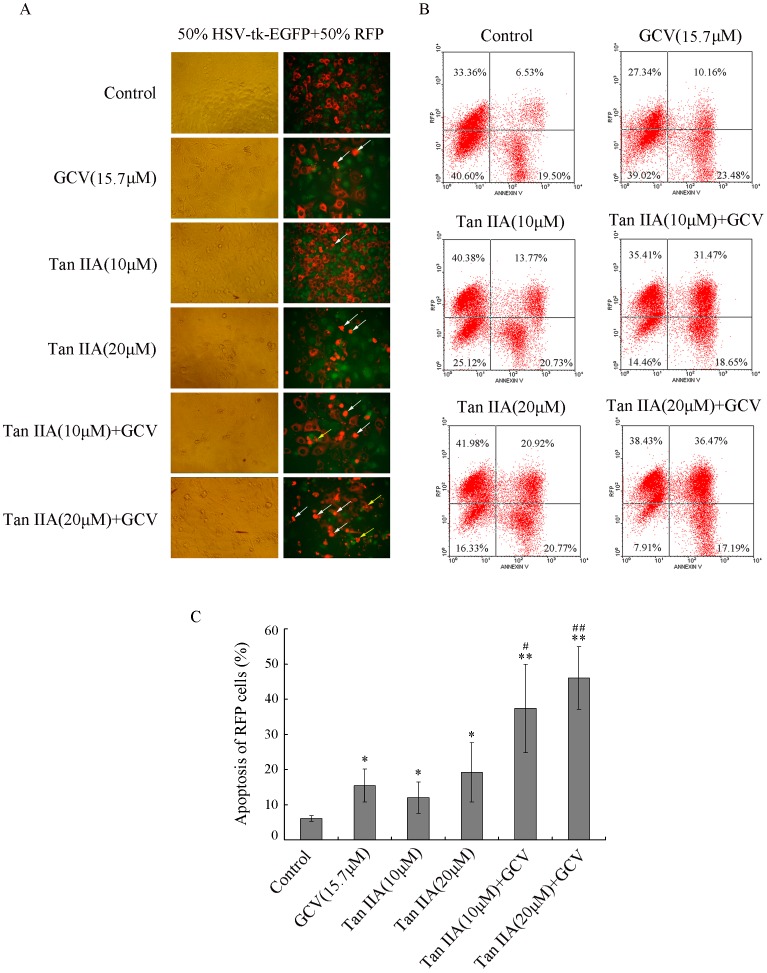
Tan IIA enhances the bystander effect. Stable HSV-tk-EGFP and RFP B16 cell lines were mixed at a ratio of 1∶1. The mixture of cells was left untreated or treated with Tan IIA (10 or 20 µM) for 24 h, and then was cultured with or without GCV (15.7 µM) for 48 h. (A) Representative images as shown by fluorescence microscopy. The red fluorescence in living RFP cells mainly localizes in cytoplasm; the aggregation of red fluorescence indicates cell shrinkage which is the hallmark of apoptosis (white arrows); the clustered light spots indicate the formation of apoptosis bodies (yellow arrows). (B) The apoptosis of RFP cells was analyzed by flow cytometry with annexin V stain. (C) Quantification of three independent experiments. *p<0.05, **p<0.01 compared with control; ^#^p<0.05, ^#^p<0.01 compared with Tan IIA or GCV treatment alone.

GJIC directly contributes to the bystander effect of the HSV-tk/GCV system for gene suicide therapy. To investigate the bystander effect of Tan IIA on B16 cells, a cell mixing assay was performed in which HSV-tk-EGFP-expressing cells (green) and RFP-expressing cells (red) were mixed at a ratio of 1∶1. After treatment with Tan IIA (0, 10 and 20 µM) for 72 h in the absence or presence of GCV (15.7 µM), the mixed cells were observed by fluorescence microscopy. As seen in Figure 3A, when cells were treated with Tan IIA or GCV treatment alone, only a small proportion of FRP cells underwent apoptosis (the aggregation of red fluorescence). In comparison, the combinatorial treatment of Tan IIA and GCV resulted in the remarkably increased proportion of apoptosis. To quantify these results, flow cytometry with annexin V stain was performed. The upper right quadrant indicates the apoptotic RFP cells (RFP and annexin V double-positive cells). As seen in Figure 3B, GCV or Tan IIA (10 and 20 µM, respectively) treatment alone resulted in only a small proportion of apoptosis of RFP cells (15.4%, 12.0% and 19.2%, respectively) (*p<0.05; **p<0.01). In contrast, the percentage of apoptosis of RFP cells was remarkably increased when cells were cultured with Tan IIA and GCV together (37.4% and 46.1%, respectively) (^##^p<0.01). The combination effect of drugs was assessed with Q value (2.12 and 1.88, respectively), which showed synergism (Q >1.15). These results suggested that Tan IIA enhances the HSV-tk/GCV-mediated bystander effect.

**Figure 4:**
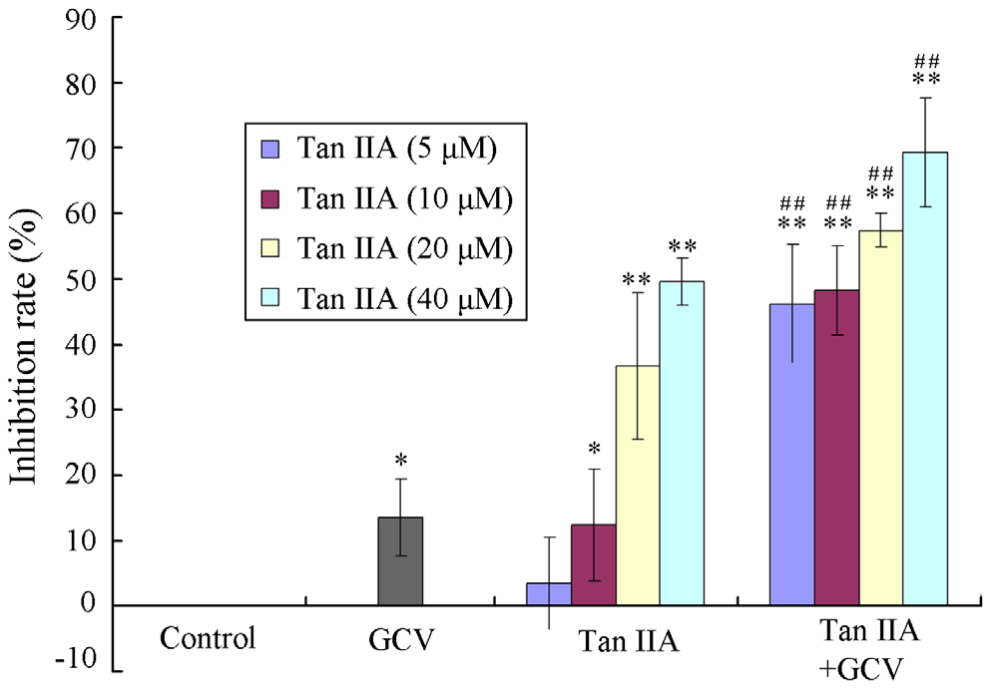
Tan IIA in combination with GCV causes greater growth inhibition on a mixture of HSV-tk and WT B16 cells. HSV-tk B16 cells were mixed with WT B16 cell line at a ratio of 1∶9. The mixture of cells was left untreated or treated with Tan IIA (10 or 20 µM) for 24 h, and then was cultured with or without GCV (15.7 µM) for 48 h followed by MTT assay. *p<0.05, **p<0.01 compared with control; ^##^p<0.01 compared with Tan IIA or GCV treatment alone.

To confirm the bystander effect of Tan IIA on B16 cells, an MTT assay was performed to assess cell viability for HSV-tk cells and WT B16 cells combined at a ratio of 1∶9. Cells were treated with Tan IIA (0, 5, 10, 20 or 40 µM) for 72 h in the absence or presence of GCV (15.7 µM). As seen at Figure 4, after GCV treatment alone, only a small inhibition of viability was observed. Although Tan IIA treatment had some cytotoxicity for the cells (3.5%, 12.3%, 36.7% and 50.6%) (*p<0.05), Tan IIA together with GCV caused greater inhibition of mixed cells (46.2%, 48.3%, 57.4% and 69.4%, respectively) (^##^p<0.01). The combination effect of drugs was assessed with Q value (2.79, 1.99, 1.15 and 1.21, respectively), which showed synergism (Q >1.15) between Tan IIA and GCV on growth inhibition of B16 cells.

**Figure 5:**
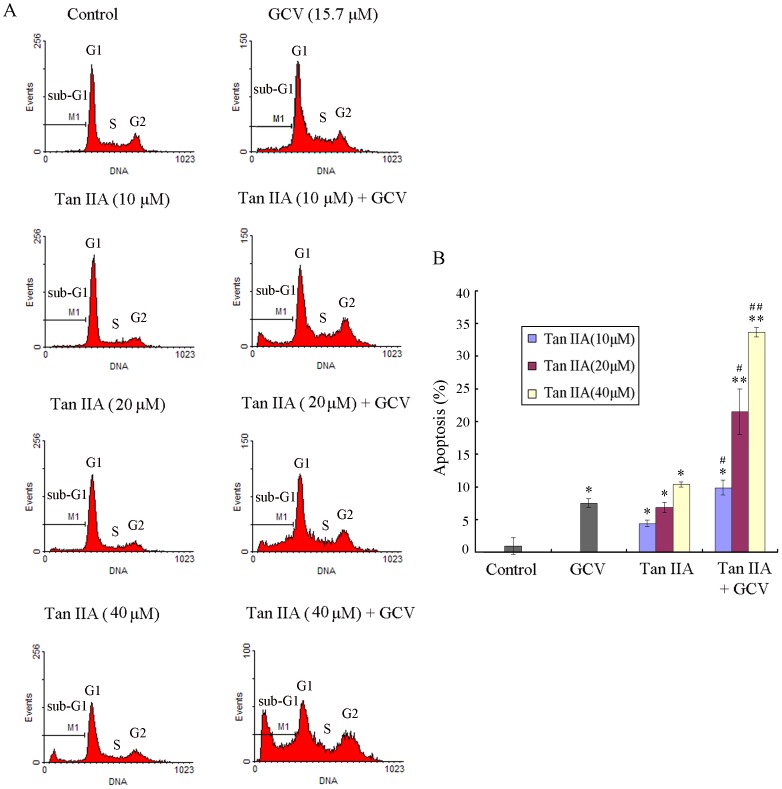
Tan IIA synergizes with GCV to promote the apoptosis for a mixture of HSV-tk and WT B16 cells. HSV-tk B16 cells were mixed with WT B16 cells at a ratio of 1∶9. The mixture of cells was left untreated or treated with Tan IIA (10 or 20 µM,) for 24 h, and then was cultured with or without GCV (15.7 µM) for 48 h. (A) Representative images of cell cycle as assessed by flow cytometry with PI stain. (B) Quantification of three independent experiments. *p<0.05, **p<0.01 compared with control; ^#^p<0.05, ^##^p<0.01 compared with Tan IIA or GCV treatment alone.

Furthermore, flow cytometry with PI stain was performed to analyze the apoptosis of a 1∶9 mixture of the HSV-tk and WT B16 cells. As seen in Figure 5, GCV or Tan IIA (10, 20 and 40 µM, respectively) treatment alone resulted in only a small percentage of the mixed cells at sub-G1 phase which indicates apoptosis (7.5%, 4.4%, 6.9% and 10.4%, respectively) (*p<0.05). When cells were treated with the combination of Tan IIA and GCV, the proportion of cells at sub-G1 phase remarkably increased to 9.8%, 21.5% and 33.6%, respectively (^#^p<0.05). The combination effect of Tan IIA and GCV was assessed with Q value (1.15, 1.39 and 1.92, respectively), which showed synergism (Q >1.15).

Altogether, these results demonstrate that Tan IIA augments the in vitro effects in the HSV-tk/GCV suicide gene system, substantiating the bystander effect as a mechanism for Tan IIA enhancement.

### In vivo Analysis of the Bystander Effect

**Figure 6:**
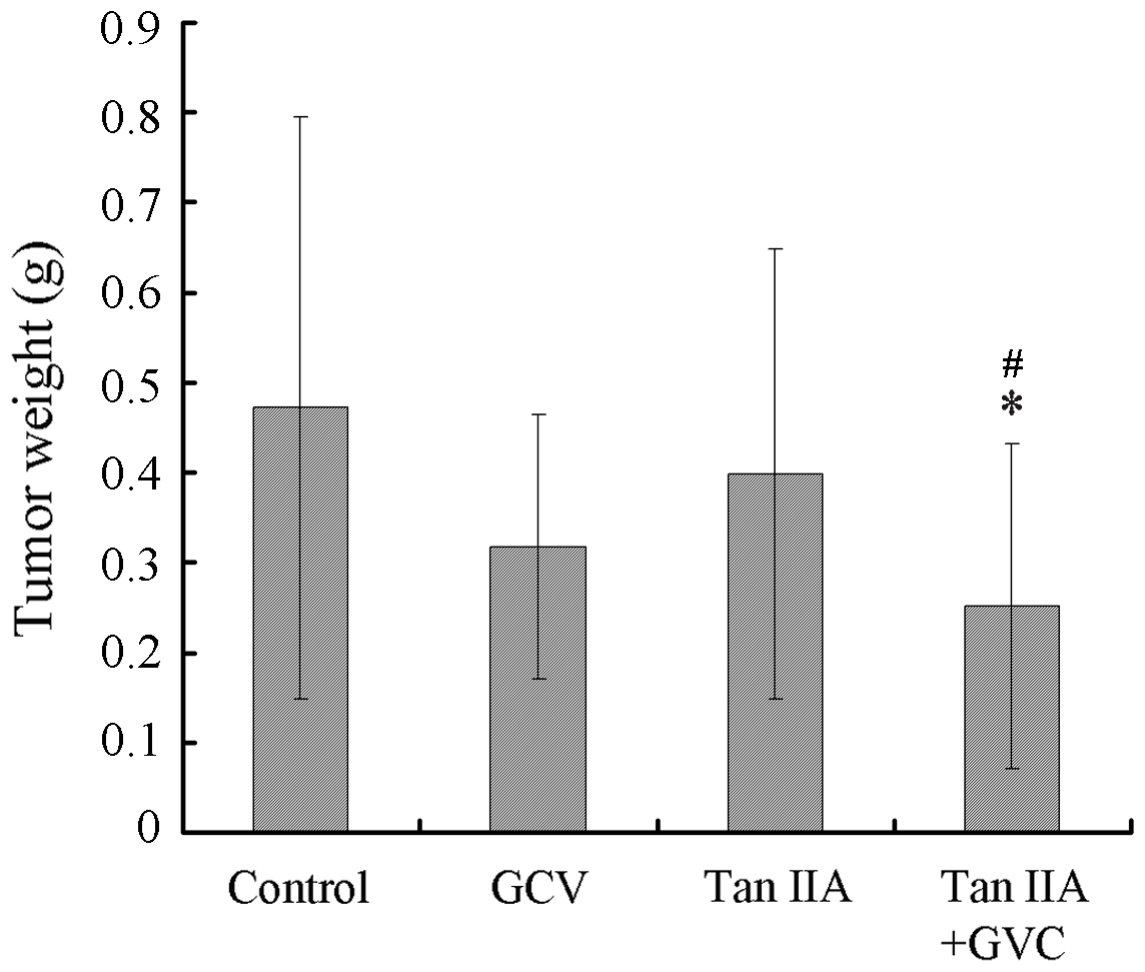
The combination effects of Tan IIA and GCV on tumor formation for a mixed population of HSV-tk and WT B16 cells in vivo. Subcutaneous B16 tumors were established in C57BL/6J mice using a 1∶9 mixture of HSV-tk and WT B16 cells. Mice were then divided into four treatment groups: control (saline only), Tan IIA only, GCV only, and Tan IIA plus GCV. The tumor weight was measured on 14 days following treatment. *p<0.05 compared with control; ^#^p<0.05 compared with Tan IIA treatment alone.

To verify these encouraging in vitro results, we examined the effects of Tan IIA on the in vivo HSV-tk/GCV bystander effect. A mixture of 10% HSV-tk B16 cells and 90% WT B16 cells were injected into each of 80 mice to produce subcutaneous B16 tumors containing cells of mixed origins. Mice were then divided into four treatment groups: control (saline only), Tan IIA only, GCV only, and Tan IIA plus GCV. The tumor weight was measured on 14 days following treatment. Figure 6 shows that mice treated with GCV alone had a 32.0% reduction in tumor weight (0.32 g as compared with 0.47 g for the control, p>0.05), mice treated with Tan IIA alone had only a 15.9% reduction in tumor weight (0.40 g, p>0.05 compared with control). Notably, mice treated with both GCV and Tan IIA caused a 46.8% reduction in tumor weight (0.25 g, *P<0.05 compared with control; ^#^p<0.05 compared with Tan IIA treatment alone). Collectively, these data demonstrate that Tan IIA synergizes with GCV to inhibit the growth of tumor cells in vitro, and that an increase in tumor inhibition may also occur in vivo via the enhancement of the HSV-tk/GCV bystander effect mediated by GJIC.

## Discussion

Gene therapy delivery protocols have been limited by the inefficiency of the systems to deliver therapeutic genes effectively to a large proportion of the tumor cell population. Consequently, there is a necessity to increase the access of cytotoxic agents to a greater amount of the tumor mass. The bystander effect offers a way to access a larger cancer cell population by allowing transportation of the activated anticancer agent to adjacent cells that were not originally targeted. Although the mechanisms underlying the bystander effect remain controversial, previous studies have clearly indicated that metabolic cooperation, mediated by gap junctions, is a major contributor to the phenomenon [8], [31]. The current study supports this hypothesis and further demonstrates a means of improving the efficiency of the bystander effect by treatment with Tan IIA to induce Cx26 and Cx43 and GJIC.

Tan IIA was originally extracted from Danshen, which a highly valued plant whose roots are used in traditional Chinese medicine to treat cardiovascular diseases [23]. It is well documented that Tan IIA also possesses anti-inflammatory activities [24] and anti-oxidant properties [25]. Additionally Tan IIA has gained attention for its anticancer effects both in cell culture and animal carcinogenesis models [26], [27]. Previously, Tan IIA was reported to restore Cx43 protein by depressing the elevated miR-1 level in ischemic and hypoxic cardiomyocytes [28]. We also demonstrated that expression of Cx26 and Cx43 is increased by Tan IIA in melanoma cells, whereas no change was observed in Cx30. Because connexins play important roles in intercellular communication, we further examined the GJIC of melanoma cells treated with Tan IIA. Double-dye transfer experiments indicated the significant enhancement of GJIC between B16 cells following Tan IIA treatment, which could be a result of the increased expression of Cx26 and Cx43.

GJIC is known to play a role in the regulation of carcinogenesis. Reduced or aberrant gap joint formation or connexin expression has been found in many tumors [32], [33], [34]. The induction of connexin expression is known to affect GJIC and gap joints may act as tumor suppressors [35], [36], [37]. Connexin transfection and GJIC induction lead to a decreased rate of proliferation, increased differentiation, and reversal of the cell-transformation phenotype [14], [15]. GJIC also plays important role in bystander cell death seen in HSV-tk gene therapy [38]. A number of chemicals such as all-trans retinoic acid, cyclic-AMP and retinoid have been reported to augment bystander effect in HSV/tk suicide gene therapy via induction of GJIC [39], [40], [41]. Since Tan IIA enhanced GJIC between B16 cells by induce upregulation of Cx26 and Cx43 expression. We speculate that Tan IIA may enhance the bystander effect with GCV treatment in cancer cells. Our data support the hypothesis that Tan IIA increases the bystander effect both in vitro and in vivo.

In conclusion, we demonstrated that Tan IIA increases Cx26 and Cx43 expression in B16 cells, and that Tan IIA also increases GJIC. Furthermore, Tan IIA was determined to augment the bystander effect in B16 tumor cell lines transduced with HSV-tk. This augmentation of the bystander effect was also supported by in vivo studies.

## Supporting Information

Figure S1 Tan IIA treatment of B16 cells results in the upregulation of Cx26 and Cx43 proteins. B16 cells were treated with Tan IIA (0, 5, 10 or 20 µM) for 24 h. (A) Immunoblotting was performed using antibody against Cx26, Cx30 and Cx43. Actin was also tested as a loading control. (B) Relative quantification of the immunoblotting results as calculated by gray scanning (*p<0.05). The results shown were representative of three independent experiments.
(TIF)
