# Supplementary figures and images for: Tanshinone IIA Increases the Bystander Effect of Herpes Simplex Virus Thymidine Kinase/Ganciclovir Gene Therapy via Enhanced Gap Junctional Intercellular Communication
Source: PLoS One. 2013 Jul 4;8(7):e67662. doi: 10.1371/journal.pone.0067662 (PMC3701623; doi:10.1371/journal.pone.0067662)

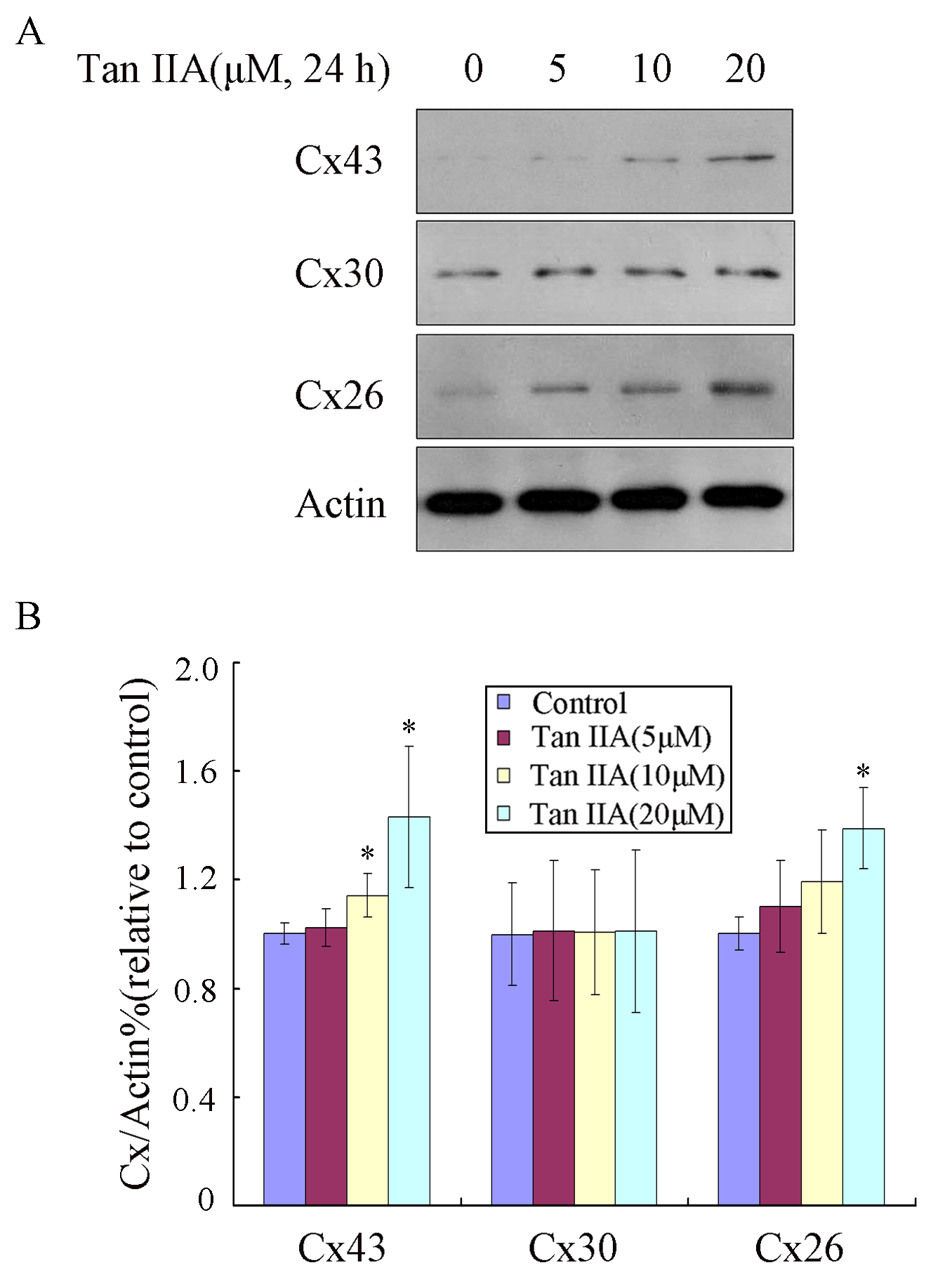

Supplement: Figure S1 — Tan IIA treatment of B16 cells results in the upregulation of Cx26 and Cx43 proteins. B16 cells were treated with Tan IIA (0, 5, 10 or 20 µM) for 24 h. (A) Immunoblotting was performed using antibody against Cx26, Cx30 and Cx43. Actin was also tested as a loading control. (B) Relative quantification of the immunoblotting results as calculated by gray scanning (*p<0.05). The results shown were representative of three independent experiments. (TIF) [file pone.0067662.s001.tif]
